# 

**Published:** 1877-12

**Authors:** 


### HALL'S JOURNAL OF HEALTH.

E. H. GIBBS, M.D., Editor.

Vol. XXIV.] December, 1877. [No. 12.

### OUR QUARTER-CENTENNIAL YEAR.

Hall's Journal of Health enters next month upon its twenty-fifth year. Its first number left the printer's hands in December, 1853. Since that period it has never omitted an issue. It was the first health journal for the people ever published, and for many years it was the only one. It has outlived a multitude of publications intended for the instruction of mankind in matters relating to their physical well-being. It has prospered abundantly, while others have dwindled and died. Its success is to be attributed to the fact that it has ridden no hobbies, advocated no single system. It has told its readers thousands of times that hygiene did not mean eating bran-bread as a staple ; that water was not made exclusively to soak people in ; that all the ills which flesh is heir to are not to be cured by the bottled fluids of the patent-medicine man, however artistic may be the label, or however pretentious the language upon it. It has told people how to live, how to eat, how to drink, how to dress, how to exercise, and how to behave, and how not to take medicine, so as to enjoy the highest measure of health and happiness.

At the close of the year we send out some thousands of copies of the Journal of Health as a free gift to prominent citizens in various localities, whose names are not now on our subscription lists. Many of these we hope to number among our subscribers for 1878. We should feel quite sure of enrolling them if we could induce them to carefully peruse the articles in this issue. Let us make a bargain : We will give the copy of our Magazine, and you, to whom we present it, shall pledge yourself to read EVERY WORD WHICH IT CONTAINS ! Then, if you believe twelve numbers, as good or better, will be worth twelve and a half cents each, you shall remit us $1.50, and receive the Journal regularly throughout the year ; or, you shall send us an even dollar, and we will mail you the Journal eight months.

The Health Food Company, No 74 Fourth Avenue, corner of Tenth Street, New York, and Mr. F. Edwards, 166 and 168 Atlantic Avenue, Brooklyn, have kindly consented to receive subscriptions, and are duly authorized to receive money intended for us.

### NEW BOOKS.

Messrs. Lee & Shepard send us four beautiful books, all admirably adapted for holiday gifts: "Ballads of Bravery," a compilation of the best poems illustrative of human heroism, with forty well-executed cuts—a worthy present for young or old; "Just his Luck"—a sea story full of incident and wonderfully attractive to boys. "His own Master,"—one of Trowbridge's best—interesting to all; "Child Marian Abroad"—a delightfully entertaining history of the doings of a bright little sight-seer; just what all little girls would like to read.

### *Index to "Hall's Journal of Health" for the Year 1877. Vol. 24.*

A — Page

A Sick Mind, 48
Annual Ailments 64
A Dangerous Curiosity 110
Agriculture 159
A Surfeit 204
A Filthy Atmosphere 262
Avenues of Death 271
A Lesson to Parents 303

B

Brain Workers 337
Baths and Bathing 112, 129
Bare Neck and Arms 113
Beautiful Old Age 141
Bartholdi's Colossal "Liberty" 177
Beautiful Hair 226
Bowel Complaints of Children 292

C

Consumption 1, 295
Cold Baths 11
Civilization and Health 38
Consumption Cure 43
Coffee Drinking 69
Common Sense 97
Constipation 124, 233, 326
Children Eating 126
Checked Perspiration 151, 235
Care of the Feet 166
Count Golfalioneri 197
Care of the Eyes 197
Cure for Drunkenness 206
Cold Feet 227
Crying Babies 227
Coughing in Consumption 241
Consumption—A Suggestion 247
Cure of Corns 273
Celibacy 316
Carelessness 318
Cancer Cured by Electricity 326

D

Dr. Hall's Spirometer 343
Drinking at Meals 3
Disease and Benevolence 33
Digestion 79
Drinking and Death 132
Dangers of Spring 134
Damp Walls 199
Death's Weapons 270
Daring to Do 291

E

Equanimity of Mind 47
Exercise 169
Early Marriages 194

F

Farmers and Citizens 105
Fruits in Summer 195
Face Painting 261
Frost Work 306
Flower Pots 334

G

Greed of Gold 289

H

Hot Air Furnaces 341
Healthy Employments 19
Human Hope 73
Heart Affections 78
Habit 96
Health of Children 171
Health Rules 188
Heart Disease 201
Hearty Suppers 275
Health and Fun 302
Hunger 342

I

Insanity 35
Improving Time 149
Influence 158
Ignorance and Ill Health 243
Incurable Insanity 246
Inhalation and Consumption 314

K

Kindness the Best Punishment 244

L

Laughter and Music 269

Page

Life and Death 212
Living Beyond One's Means 229
Leaden Water Pipes 233
Life Insurance 61, 178
Liquor Drinking 133
Living Ages 142
Lead Poison 80

M

Marvels of Man 93
Marrying Well 255
Marriage in England 280
Maccaroni 313

N

Never 18
Nature's Methods 330

O

Out-Door Safety 52
Over Eating 107
Object of Eating 143
Our Proverbs 253

P

Politics and Physic 5
Practical Knowledge 44
Pure Air in Medicine 70
Pestilence and Plague 115
Punishing Children 163
Popular Fallacies 166
Parental Corrections 181
Preventing Disease 289

Q

Quenching Thirst 132

R

Reforming a Reformer 144
Remedy for a Cough 153
Reason in all Things 175
Rage and Ruin 243
Reading 319

S

Shaving the Beard 329
Sprains 77
Small-Pox 100
Sleeping Rooms 104
Sleeping Position 114
Sudden Death 130
Second Childhood 136
Sorrowing Poverty 139
Spring Ailments 148
Sores 200
Sleeping Together 202
Spring Disease 125, 204
Science Aiding Justice 218
Summer Complaints of Children 237
Sea Sickness 259
Sun Baths 306

T

To Stop a Cough 324
To Bring up Children 17
Throat-Ail Causes 21
Tube Wells 339
The Literature of the Times 74
The Language of Flowers 84
The Teeth 102
The Feet in Winter 111
The Grave of Hope 121
Temperament Differences 127
Throat-Ail 156
The Eyes 148, 206
The Instinct of Appetite 212
The Achievements of Science 215
The Best Inheritance 221
To Stop a Cough 324
The Nice Young Man 225
The Little Courtesies of Life 239
The Secret Out 270
Trials of Life 278
The Expression of Dress 283

V

Ventilation 99, 335
Valuable Table 246

W

Weakly Youths 140
Walk Softly 140
Who are Happiest? 284

